# Linking auditory brain responses to cortical microstructure and sensory behaviors in autism spectrum disorder: a preliminary study

**DOI:** 10.3389/fpsyt.2026.1688324

**Published:** 2026-07-24

**Authors:** Natsuko Kashida, Kazuhiko Yamamuro, Kiwamu Matsuoka, Ryou Mizui, Rio Ishida, Tsutomu Takeda, Hiroto Tamakoshi, Takahiro Yoshihara, Masato Takahashi, Takahira Yamauchi, Michihiro Toritsuka, Nakao Iwata, Manabu Makinodan

**Affiliations:** 1 Department of Neuropsychiatry, Kumamoto University School of Medicine, Kumamoto, Kumamoto, Japan; 2 Center for Health Control, Nara Medical University, Kashihara, Nara, Japan; 3 Department of Psychiatry, Nara Medical University School of Medicine, Kashihara, Nara, Japan; 4 Matsuoka Clinic, Kashihara, Nara, Japan; 5 Department of Psychiatry, Fujita Health University School of Medicine, Toyoake, Aichi, Japan

**Keywords:** auditory brainstem response, autism spectrum disorder, brain microstructure, cognitive inflexibility, sensory hypersensitivity

## Abstract

**Introduction:**

Atypical auditory processing is a core characteristic of autism spectrum disorder (ASD), potentially stemming from disrupted thalamocortical circuits and frontal modulation. This study investigated whether individual differences in cortical microstructure, as measured by neurite orientation dispersion and density imaging, are associated with auditory brainstem responses (ABR) and whether these ABR measures are associated with autism traits and atypical sensory processing.

**Methods:**

We recruited 15 adults with ASD (9 males, 6 females; mean age 26.9 ± 6.7 years) and 12 typically developing controls (12 males; mean age 37.1 ± 8.0 years) and assessed microstructural properties in the thalamus, temporal cortex, and orbitofrontal cortex (OFC). Auditory processing was evaluated via ABR recorded under forward-masking conditions.

**Results:**

In this preliminary, exploratory analysis, mediation models suggested that the amplitude of wave PVII mediated the association between the orientation dispersion index in the temporal cortex and autism traits, as measured by the Autism-Spectrum Quotient. Similarly, the ΔVI amplitude (peak-to-peak potential between NVI and PVI) mediated the relationship between the neurite density index in the OFC and atypical sensory behaviors, assessed using the Adolescent/Adult Sensory Profile. In the ASD group, reduced PVII amplitude was linked to difficulties in attention switching and imagination, while increased ΔVI amplitude was associated with sensory avoidance.

**Discussion/conclusion:**

Our preliminary and exploratory findings tentatively suggest that microstructural variability in the temporal cortex and OFC may relate to auditory neural responses and to sensory and cognitive features of ASD. However, given the small and demographically imbalanced sample, these results should be regarded as hypothesis-generating rather than confirmatory. These candidate brain–behavior pathways should be tested in larger, demographically matched cohorts before any mechanistic interpretation regarding sensory dysfunction in autism can be drawn.

## 1 Introduction

Atypical auditory processing is a core feature of autism spectrum disorder (ASD) and has been linked to abnormal thalamocortical connectivity (1–13). The thalamus, composed of multiple nuclei, serves as a critical relay structure within the auditory system, while the temporal cortex is responsible for auditory recognition and multisensory integration (14–19). The prefrontal cortex plays a pivotal role in auditory cognition by receiving input from auditory association areas and multisensory regions such as the superior temporal sulcus (20–22). The inhibitory thalamic reticular nucleus (TRN) intercepts and modulates all corticothalamic and thalamocortical communications, serving as a key regulator of thalamic output to the cortex. Among the subregions of the prefrontal cortex, the orbital area, specifically Area 13, has been shown in macaque studies to give rise to broadly distributed projections within the TRN, overlapping with TRN terminal fields originating from the auditory cortex of the temporal lobe (23).

Recent research suggests that reduced thalamic inhibition (i.e., suppression of incoming auditory input) in ASD may lead to increased perception of auditory input and impaired auditory filtering, thereby contributing to attention difficulties and auditory hypersensitivity (24). Evidence from genetics, animal models, neuroimaging, and electrophysiology studies supports the involvement of thalamocortical circuit dysfunction in the pathophysiology of ASD. A study using a genetic mouse model has demonstrated that impaired auditory filtering compromises attention and increases sensory sensitivity (25). These findings underscore the importance of the thalamus and temporal cortex in complex auditory processing.

Individuals with ASD demonstrate a reduced ability to filter background noise (26–29). Mamashli et al. (30) used magnetoencephalography to examine auditory responses in individuals with ASD and found that the ASD group tended to show weaker evoked mismatch fields under noisy conditions, suggesting that atypical auditory responses may reflect auditory hypersensitivity and altered language processing. In addition, disrupted functional connections between the temporal and frontal cortices suggest impaired top-down control from the temporal gyrus to the inferior frontal gyrus. Previous studies using forward-masking auditory brainstem response (ABR) have demonstrated a diminished response (i.e., reduced ABR amplitude) when preceded by masking sounds and decreased thalamic activity in attention deficit hyperactivity disorder (31, 32). These observations highlight the need to investigate not only the thalamus and temporal cortex but also frontal brain structures in ASD.

Children with ASD are more likely to experience multiple adverse childhood experiences (ACEs), such as neglect, abuse, or household dysfunction, which can have lasting effects on psychological and neurodevelopmental outcomes (33–35). Structural abnormalities in the frontal cortex have been associated with childhood trauma and adversity, with magnetic resonance imaging (MRI) studies showing alterations in frontal lobe volume (36–39). In our previous study, we used neurite orientation, dispersion, and density imaging (NODDI) to quantify axonal and dendritic density (neurite density index [NDI] and orientation dispersion index [ODI]) and found a significant positive correlation between post-traumatic stress disorder (PTSD) symptom severity and frontal NDI in individuals with ASD. This association was specifically observed in those who had reported ACEs (40). The orbitofrontal cortex (OFC) has strong connections with limbic structures and is involved in emotional processing (21). Moreover, stress during development induces alterations in dendritic morphology in the OFC (41). These findings raise the possibility that adversity is associated with frontal microstructure and atypical sensory processing.

Atypical auditory filtering may be relevant not only to auditory hypersensitivity but also to broader autistic traits. Difficulties in filtering or integrating sensory input can affect attention allocation, cognitive flexibility, and the interpretation of socially relevant environmental cues, processes closely related to core ASD characteristics (24, 26–30). In particular, thalamocortical circuits and temporal–frontal networks are involved not only in sensory transmission but also in higher-order processes such as attentional control and multisensory integration (14–23). Therefore, individual differences in these neural pathways may be associated with autistic traits, including difficulties with attention switching and imagination, as assessed by the Autism-Spectrum Quotient. Given the limited prior work directly linking NODDI-derived cortical microstructure to brainstem auditory responses in ASD, the present study was designed as an exploratory, hypothesis-generating investigation rather than as a confirmatory test of a pre-specified model. Within this exploratory framework, we examined whether individual differences in the microstructural properties of the thalamus, temporal cortex, and OFC, as measured by NODDI, were associated with ABR under forward-masking conditions and with atypical sensory processing and autistic traits. We further examined whether ABR measures mediated the relationships between regional microstructural indices and these behavioral outcomes.

## 2 Methods

### 2.1 Participants

The present study involved 15 patients with ASD (9 males, 6 females; mean age 26.9 ± 6.7 years) and 12 typically developing (TD) controls (12 males; mean age 37.1 ± 8.0 years). All participants were Japanese, born and residing in Japan. Patients with ASD were recruited from the outpatient services of the Department of Psychiatry, Nara Medical University Hospital, and its affiliated psychiatric clinic. Two trained psychiatrists diagnosed ASD using the criteria from the Diagnostic and Statistical Manual of Mental Disorders, 5th edition (DSM-5), which were further validated by trained psychiatrists and psychologists using the Autism Diagnostic Observation Schedule, 2nd edition (ADOS-2) (42, 43). Participants in the TD group were recruited through local print advertisements. They had no history of psychiatric illness, neurological illness, or developmental disorders and were asked to complete a mini-international neuropsychiatric interview to rule out a current or past history of psychiatric illness. In addition, the Autism Spectrum Index, Japanese version (AQ-J) was used to evaluate patients; a score of less than 27 was required for enrollment (44). Exclusion criteria for both groups included neurological disease, head injury, serious medical conditions, and a history of substance abuse/dependence. This study was approved by the Ethics Committee of Nara Medical University and was conducted in accordance with the Declaration of Helsinki. Written informed consent was obtained from all participants prior to study participation.

### 2.2 Measures

#### 2.2.1 Autism traits

Autism traits were assessed using the AQ-J, a self-report questionnaire measuring autism-like characteristics in adults with average or above-average intelligence. This 50-item questionnaire measures autism traits across five subdomains, namely social skill, attention switching, attention to detail, communication, and imagination. Each item is scored on a 4-point Likert scale with the following response options: “definitely agree,” “slightly agree,” “slightly disagree,” and “definitely disagree.” The AQ-J has been previously validated and is widely used (45, 46).

#### 2.2.2 Atypical sensory processing

Atypical sensory processing characteristics were assessed using the 60-item Adolescent/Adult Sensory Profile (AASP), a self-report questionnaire evaluating individuals’ responses to everyday sensory experiences (47, 48). Items are rated on a 5-point Likert scale, ranging from 1 (Almost Never) to 5 (Almost Always) and are organized into four atypical sensory processing patterns: low registration, sensory seeking, sensory sensitivity, and sensory avoiding. The AASP is grounded in Dunn’s theoretical model of atypical sensory processing, which conceptualizes sensory responses based on neurological thresholds and behavioral responses (49). Higher pattern scores reflect greater deviations in the respective domain.

#### 2.2.3 Trauma history

We used the Japanese version of the Child Abuse and Trauma Scale (CATS) to assess trauma history (50). The CATS is a 38-item instrument that retrospectively evaluates ACEs (51). Each item is measured on a 5-point scale, ranging from 0 (never) to 4 (always) and divided into five main factors of ACEs, including neglect/negative home atmosphere, sexual abuse, punishment, emotional abuse, and other. Total scores are calculated by adding the scores across all 38 items and can range from 0 to 152. Higher total scores indicate greater perceived severity of childhood abuse and trauma.

The severity of PTSD symptoms was measured using the Japanese version of the Impact of Event Scale-Revised (IES-R-J) (52). The IES-R-J is commonly used to evaluate self-reported posttraumatic stress symptoms, such as intrusion (eight items), hyperarousal (six items), and avoidance (eight items), after a traumatic experience. Participants rated each questionnaire item with respect to one past life event that forced them to feel great discomfort on a scale of 0 (not at all) to 4 (extremely); the IES-R-J total and subscale (intrusion, hyperarousal, and avoidance) scores were calculated accordingly, with higher scores reflecting greater severity of PTSD symptoms.

### 2.3 ABR

#### 2.3.1 Stimuli and apparatus

**Figure 1:**
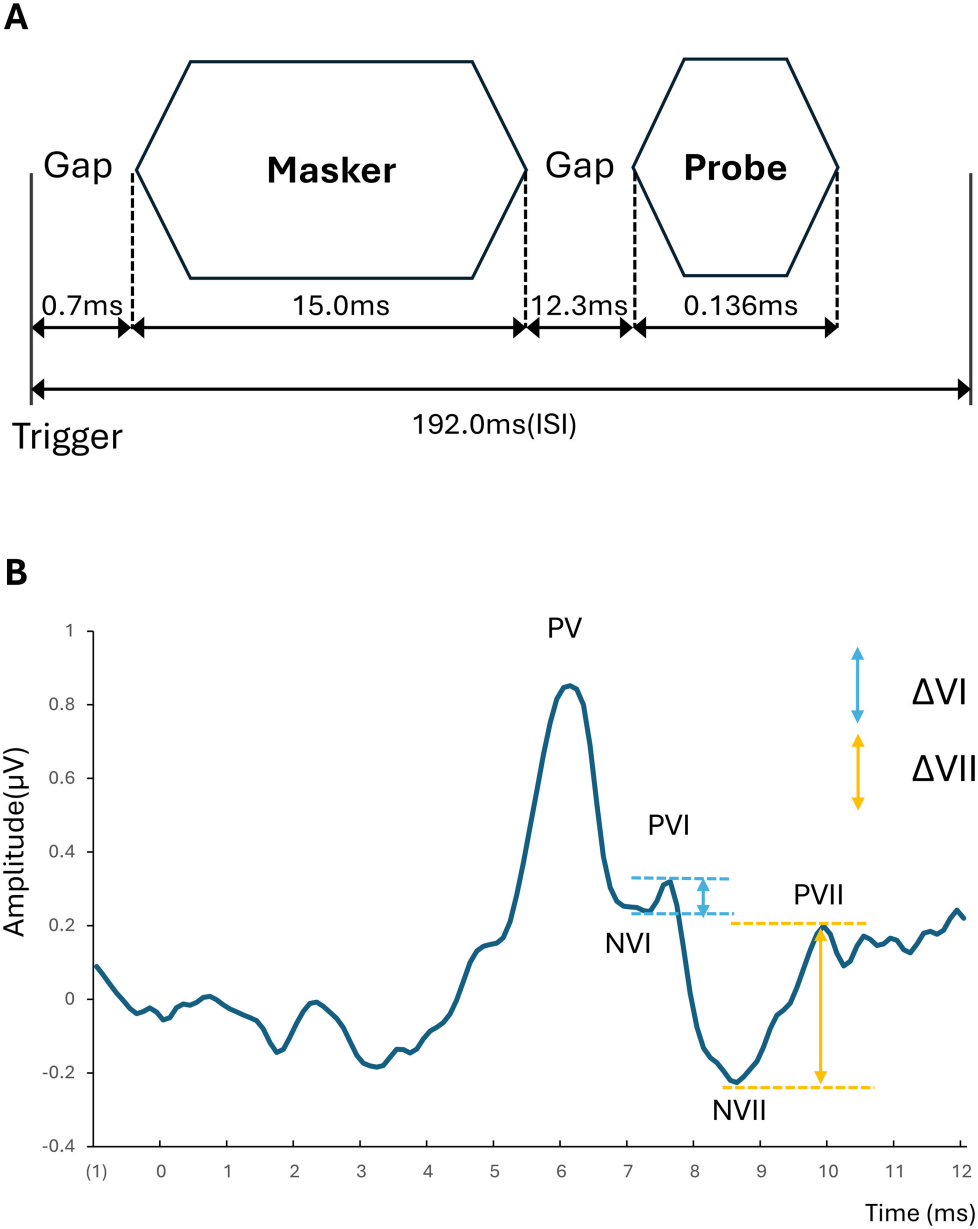
Auditory brainstem response (ABR) under forward masking conditions. **(A)** Schematic representation of the forward masking auditory stimulus. The masker consists of 1,500 Hz low-pass filtered noise; the probe is a rectangular click pulse. ISI, inter-stimulus interval. **(B)** Representative ABR waveform recorded at the P3 electrode site in an individual with ASD. NVI, negative peak preceding PVI; PVI, positive peak of wave VI; NVII, negative peak preceding PVII; PVII, positive peak of wave VII; ΔVI, peak-to-peak amplitude between NVI and PVI (blue); ΔVII, peak-to-peak amplitude between NVII and PVII (yellow).

ABR was recorded using EEG-1260 (Japan Kohden, Tokyo, Japan). Binaural auditory stimuli were delivered via earbuds using the Multi Trigger System (Medical Try System, Japan). Stimuli were presented at an 80 dB sound pressure level, repeated 2,048 times. A rectangular click pulse was used as a probe in the forward masking paradigm. The probe had a duration of 0.136 ms with rise and fall times of 0.023 ms. Stimuli were delivered at an inter-stimulus interval of 0.192 s. The masker preceding the click was 1,500 Hz low-pass filtered noise (Butterworth filter) with a duration of 0.015 s, including 0.004 s rise and fall times. The interval between the masker and the target stimulus was 0.0123 s (**Figure 1A**). The onset-to-onset interval between the click and the forward masker stimulus was 0.192 s. All auditory stimuli were generated using DigiOnSound (DigiOn, Inc., Japan). These stimulus parameters were based on the method described by Källstrand and colleagues (31). A schematic illustration of this forward-masking paradigm is provided in **Figure 1A**.

### 2.4 Procedure

All recordings were conducted in a quiet room. Participants were seated comfortably in armchairs and instructed to relax in a resting state. Scalp potentials were recorded on 15 Ag/AgCl electrodes mounted in an elastic cap (Wavegard Cap) according to the extended 10–20 system. The ground electrode was located at FPz, and the system reference was at C3 and C4. Electrode impedance and inter-electrode impedance were confirmed to be below 5 kΩ both before and after the recordings. Participants were instructed to close their eyes and relax and were allowed to fall asleep. Apart from listening to the auditory stimuli, no other active task was demanded of the participants. Prior to testing, the participants were verbally informed of the procedure.

### 2.5 Analysis

ABR data were analyzed using EMSE561 software (CORTECH SOLUTIONS, INC. America). Active electrodes were P3 and P4, with the earlobe serving as the reference. Continuous electroencephalography signals were band-pass filtered (80–1500 Hz) and notch filtered (59–61 Hz), then segmented into epochs from –1 ms to 13 ms relative to the onset of the click stimulus. Trials with excessive artifacts (e.g., high-amplitude movements) were excluded. Baseline correction was performed using a 1 ms interval preceding stimulus onset. In response to a brief click stimulus presented, the auditory brainstem pathway generates a series of electrophysiological signals that are detectable on the scalp via electroencephalography (53). Within the initial 10 ms following stimulus onset, five to seven vertex-positive waves—designated as waves I through VII—are typically observed. Additionally, a slow negative deflection emerges subsequent to wave V, beginning at approximately 6 ms. Collectively, these waveforms constitute the ABR. ABR waveforms were analyzed by identifying the positive peaks of PVI and PVII, as well as the corresponding negative peaks of NVI and NVII immediately preceding them. Peak-to-peak amplitudes were calculated for PVI–NVI (ΔVI) and PVII–NVII (ΔVII) (**Figure 1B**).

### 2.6 MRI

All MRI scans were performed using a 3.0-Tesla clinical scanner (Magnetom Skyra; Siemens, Erlangen, Germany) equipped with a 32-channel phased-array brain coil. High-resolution, three-dimensional T1-weighted images were acquired using a magnetization-prepared rapid gradient-echo sequence (repetition time [TR] = 2500 ms; echo time [TE] = 2.18 ms; inversion time = 1000 ms; field of view = 256 mm; flip angle = 8°; acquisition matrix = 320 × 300; and sagittal slices of 0.8 mm thickness). A two-shell diffusion MRI protocol was used to enable NODDI parameters. Diffusion MRIs were acquired using an echo-planar imaging sequence. Imaging parameters were as follows: TR = 3600 ms; TE = 90 ms; b = 0, 700, 2000 s/mm^2^; acquisition matrix = 120 × 120; and a flip angle of 90°. A motion probing gradient was applied in 67 anterior-posterior and 68 posterior-anterior directions.

Diffusion-weighted images were processed using Functional Magnetic Resonance Imaging of the Brain (FMRIB) software version 6.0.6.2 (FMRIB Center, Department of Clinical Neurology, University of Oxford, Oxford, England; http://www.fmrib.ox.ac.uk/fsl/) (54). Susceptibility-induced off-resonance fields were estimated, as implemented in the FMRIB Software Library (FSL) tool TOPUP, using pairs of diffusion-weighted images acquired with reversed phase-encoding blips (55). TOPUP distortion correction was applied, followed by corrections for eddy current distortions and motion using the eddy tool in FSL (56). The NODDI model was fitted using the NODDI toolbox version 1.01 (https://www.nitrc.org/projects/noddi_toolbox/) running on MATLAB 2021a (MathWorks, Natick, MA, USA). ODI and NDI value maps were calculated.

For reference regions, surface-based cortical reconstruction and volumetric subcortical segmentation of T1-weighted images were performed using the FreeSurfer software (version 7.3.2; https://surfer.nmr.mgh.harvard.edu/). The regions of interest (ROI) in the thalamus, temporal cortex, and OFC were defined (57). Each participant’s ODI and NDI value maps were co-registered to a three-dimensional T1-weighted image. Regional ODI/NDI values were calculated by averaging the values for all voxels within the ROI.

### 2.7 Data analysis

Statistical analyses were conducted using SPSS Statistics version 28 (IBM Corp., Armonk, NY) and Python 3.11 (Python Software Foundation, 2023). Normality of the distributions was assessed using the Shapiro–Wilk test, and when the assumption of normality was violated (p < 0.05), the non-parametric Mann-Whitney U test was employed. Additionally, for correlation analyses, when normality was not satisfied, partial correlation was conducted using Spearman’s rank correlation coefficient. To compare the characteristics of the participants in each group, we used Mann-Whitney’s U-test for continuous variables and the chi-square test for discrete variables.

The TD group was included as a comparison group to examine whether ABR amplitudes and NODDI-derived microstructural indices differed between individuals with ASD and those with TD. Demographic characteristics, ABR amplitudes, and NODDI indices were therefore compared between these two groups. In contrast, associations between ABR measures and ASD-related clinical characteristics, including AQ-J and AASP subscale scores, were examined within the ASD group, in line with the main objective of this study to explore brain–behavior relationships relevant to ASD. The analytic sample used for each analysis is specified in the corresponding table legends.

There were significant differences in age and sex between the TD and ASD groups. Therefore, these values were subjected to one-way analysis of covariance (ANCOVA) for each channel, with age and sex as covariates, to determine whether there were differences in ABR measurements between groups. When significant effects were found, *post hoc* comparisons were conducted using the Bonferroni correction. To investigate the associations between ABR and MRI data, as well as between ABR and psychological assessments (AQ-J, AASP, CATS, IES-R-J), partial correlation analyses were conducted while controlling for age and sex. Pearson’s partial correlation coefficients were used when the assumption of normality was met, and Spearman’s rank partial correlation coefficients were applied when normality was violated. For correlation analyses, Bonferroni correction was applied within each set of analyses according to the number of tested associations, as specified in the corresponding table legends.

All significance tests were two-tailed. For group comparisons, Bonferroni correction was applied separately to each family of tests. Specifically, ABR amplitude comparisons were corrected for 12 comparisons, corresponding to six ABR indices (NVI, PVI, NVII, PVII, ΔVI, and ΔVII) measured at two electrode sites (P3 and P4). NODDI group comparisons were corrected for six comparisons, corresponding to two NODDI indices (NDI and ODI) across three regions of interest: the temporal cortex, thalamus, and OFC. For exploratory regression and path analyses, p-values are reported as indicated in the corresponding tables, and findings were interpreted cautiously given the preliminary nature of the study. To identify predictors of ABR, a multiple linear stepwise regression analysis was conducted using ODI (Temporal, Thalamus) as the independent variable. Similarly, to identify psychological predictors of the OFC, multiple linear stepwise regression analyses were conducted using psychological assessments as independent variables and NDI and ODI in the OFC as dependent variables. Confidence intervals and *p* values were estimated using bootstrapping with 2,000 resamples. Path analysis was performed using the semopy package, a structural equation modeling library in Python (58). To evaluate the stability and significance of the estimated path coefficients, nonparametric bootstrapping (2,000 iterations) was used; two-sided *p* values and 95% confidence intervals were calculated. Of note, the path models specified in this study were just-identified (saturated), with zero degrees of freedom; under such conditions, global fit indices such as CFI, RMSEA, and χ² are mathematically constrained to indicate perfect fit and therefore cannot be used for model validation. Accordingly, fit statistics are reported for descriptive purposes only, and inference is based on the magnitude, direction, and bootstrap-based confidence of the individual path coefficients. The multiple linear stepwise regression analyses described above are considered strictly exploratory. Given the small sample size (N = 27), stepwise variable selection is vulnerable to overfitting, unstable inclusion/exclusion of predictors across resamples, and inflation of nominal significance levels. Accordingly, these analyses were not interpreted as confirmatory tests of specific predictors but rather as hypothesis-generating, and inference is anchored on the magnitude and direction of effects together with bootstrap-based (2,000 resamples) confidence intervals.

## 3 Results

### 3.1 Participant demographics and clinical characteristics

The demographic and clinical characteristics of all participants are summarized in **Table 1**. There were no significant differences in estimated IQ between the ASD and TD groups. While significant group differences were observed in age (U = 31.500, *P* = 0.004, Mann–Whitney U test) and sex distribution (χ² = 6.171, *P* = 0.013), these variables were statistically controlled in all subsequent analyses. Compared to the TD group, participants in the ASD group showed significantly higher total scores on the Japanese versions of AQ (U = 6.500, *P* < 0.001), AASP (U = 10.000, *P* < 0.001), IESR (U = 13.500, *P* < 0.001), and CATS (U = 25.500, *P* = 0.002).

**Table 1:** Demographic and clinical characteristics of participants with ASD and TD controls.

Variables	TD		ASD		Mann-Whitney U test			
	Average	SD	Average	SD	U or X^2^	Z	Adjusted *P*	r
**Age (year)**	37.083	8.028	26.933	6.745	31.500	−2.862	0.004^**^	−0.551
**Sex (male/female)**	12/0		9/6		6.171		0.013^*^	
**Estimated IQ**	104.741	9.391	99.533	14.749	68.000	−1.075	0.282	−0.207
**AQ-J total**	15.167	4.130	32.933	7.759	6.500	−4.080	<0.001^***^	−0.785
Social skill	2.083	1.832	8.067	2.404	8.500	−4.007	<0.001^***^	−0.771
Attention switching	3.000	2.000	7.133	1.846	12.000	−3.831	0.001^**^	−0.737
Local details	5.000	1.414	4.400	2.131	71.500	−0.916	1.000	−0.176
Communication	1.500	1.624	7.333	2.350	6.500	−4.120	<0.001^***^	−0.793
Imagination	3.583	2.151	6.000	2.449	41.000	−2.419	0.078	−0.465
**AASP total**	117.167	21.540	168.667	25.249	10.000	−3.905	<0.001^***^	−0.751
Low registration	22.500	5.108	42.533	8.314	1.500	−4.322	<0.001^***^	−0.832
Sensation seeking	35.917	7.856	35.000	5.542	84.500	−0.269	1.000	−0.052
Sensory sensitivity	28.833	6.860	44.800	9.073	17.000	−3.572	0.001^**^	−0.687
Sensation avoiding	29.917	7.937	46.333	10.168	17.500	−3.544	0.002^**^	−0.682
**IES-R-J total**	8.250	7.509	39.533	21.686	13.500	−3.738	<0.001^***^	−0.719
Intrusion	2.583	2.937	14.667	9.781	22.000	−3.333	0.003^**^	−0.642
Avoidance	4.750	5.754	14.867	6.937	21.000	−3.381	0.002^**^	−0.651
Hyperarousal	0.917	1.311	10.000	7.937	15.500	−3.697	0.001^**^	−0.712
**CATS total**	17.167	8.043	47.933	32.524	25.500	−3.153	0.002^**^	−0.607
Punishment	8.417	2.999	10.200	6.837	88.000	−0.098	1.000	−0.019
Neglect	4.167	4.218	18.467	12.217	18.500	−3.503	0.002^**^	−0.674
Emotional abuse	2.750	2.221	11.933	9.277	24.000	−3.239	0.006^**^	−0.623
Sexual abuse	0.000	0.000	0.000	0.000	N/A	N/A	N/A	N/A
Others	1.833	1.992	7.333	6.576	32.500	−2.842	0.022^*^	−0.547

Values represent means and standard deviations unless stated otherwise. *P* values were calculated using the Mann–Whitney U test (continuous variables) or chi-square test (categorical variables). Adjusted *p* values reflect the Bonferroni correction.

*P < 0.05, **P < 0.01, ***P < 0.001, N/A Not Applicable.

ASD, autism spectrum disorder; TD, typically developing; AQ-J, Japanese version of the Autism-Spectrum Quotient; AASP, Adolescent/Adult Sensory Profile; IES-R-J, Japanese version of the Impact of Event Scale-Revised; CATS, Child Abuse and Trauma Scale; SD, standard deviation. Bold values indicate statistically significant results or correlation coefficients with r > |0.4|.

### 3.2 Group comparison of ABR amplitudes

ABR waveforms were compared between the ASD and TD groups using one-way ANCOVA for each recording channel, with age and sex as covariates (**Supplementary Table 1**). No statistically significant group differences in ABR amplitudes were observed under forward masking conditions, suggesting comparable brainstem-level auditory responses across groups.

### 3.3 Group comparison of NDI and ODI across brain regions

Mean values of NDI and ODI were extracted for each ROI, and group comparisons were conducted using ANCOVA, with age and sex included as covariates (**Supplementary Table 2**). No statistically significant differences were observed between the ASD and TD groups in either NDI or ODI values across the temporal cortex, thalamus, or OFC. These results suggest that microstructural properties in these regions, as measured by NDI and ODI, are comparable between adults with ASD and TD.

### 3.4 Correlations between ABR amplitudes (P3/P4) and clinical or NODDI measures

**Table 2** presents the P3 and P4 amplitudes across all participants, along with their correlations with clinical assessment scores and NODDI metrics. All analyses included age and sex as covariates. No statistically significant correlations were found between psychological measures and ABR amplitudes overall. In the temporal cortex, ODI values showed significant negative correlations with both NVII (P3: r = −0.672, *P* = 0.0014; P4: r = −0.676, *P* = 0.0012) and PVII (P3: r = −0.587, *P* = 0.0122; P4: r = −0.582, P = 0.0135). In the thalamus, a significant correlation between ODI and NVII was observed at P3 (r = −0.525, P = 0.0425), but not at P4. In the OFC, significant negative correlations were found between ODI and NVII (P3: r = −0.624, *P* = 0.0051; P4: r = −0.606, *P* = 0.0079), and a trend-level association with PVII (P3: r = −0.517, *P* = 0.0491; P4: r = −0.490, *P* = 0.0770). Within the OFC, NDI values also showed a significant negative correlation with ΔVI at P3 (r = −0.571, P = 0.0174), but not at P4. These findings suggest that microstructural characteristics of the temporal cortex, thalamus, and OFC, particularly as measured by ODI and NDI, are associated with brainstem-level auditory responses in adults with ASD.

**Table 2:** Partial correlations between ABR amplitudes at P3/P4 electrode sites and clinical assessments or NODDI metrics in individuals with ASD and TD controls.

Variables	NVI		PVI		NVII		PVII		ΔVI		ΔVII	
	P3	P4	P3	P4	P3	P4	P3	P4	P3	P4	P3	P4
AQ-J total	−0.033	−0.101	−0.020	−0.040	−0.264	−0.277	**−0.442**	**−0.495**	−0.144	−0.018	**−0.411**	−0.321
AASP total	0.171	−0.047	0.297	0.090	0.230	0.227	0.256	0.227	0.348	**0.412**	0.275	0.251
IES-R-J total	0.102	−0.082	0.200	0.073	0.157	0.128	0.122	0.112	0.297	0.277	0.109	0.117
CATS total	0.072	−0.090	0.189	0.038	0.252	0.252	0.202	0.199	0.309	0.306	0.079	0.135
ODI temporal	**−0.465**	**−0.450**	**−0.481**	**−0.480**	−0.672^**^	−0.676^**^	−0.587^*^	−0.582^*^	−0.224	−0.249	−0.124	−0.087
ODI thalamus	−0.364	−0.314	−0.363	−0.309	−0.525^*^	**−0.464**	**−0.483**	**−0.453**	−0.122	−0.079	−0.137	−0.141
ODI orbitofrontal	**−0.457**	**−0.435**	**−0.467**	**−0.452**	−0.624^**^	−0.606^**^	−0.517^*^	**−0.490**	−0.199	−0.193	−0.067	−0.030
NDI temporal	0.039	0.083	−0.045	−0.018	0.010	0.046	−0.127	−0.116	−0.305	−0.340	−0.222	−0.231
NDI thalamus	−0.117	−0.149	−0.158	−0.165	**−0.447**	−0.373	−0.396	−0.361	−0.198	−0.103	−0.090	−0.109
NDI orbitofrontal	0.204	0.199	0.035	0.071	0.085	0.110	−0.025	−0.043	−0.571^*^	**−0.402**	−0.149	−0.196

Partial correlation coefficients are shown after adjusting for age and sex. Clinical assessments (Spearman’s *r*) included the AQ-J, AASP, IESR, and CATS; NODDI metrics (Pearson’s r) included regional NDI and ODI values. Values in bold indicate correlations with r > |0.4|.

*Adjusted *P* < 0.05, **Adjusted *P* < 0.01.

ABR, auditory brainstem responses; NODDI, neurite orientation, dispersion, and density imaging; ASD, autism spectrum disorder; TD, typically developing; AQ-J, Japanese version of the Autism-Spectrum Quotient; AASP, Adolescent/Adult Sensory Profile; IES-R-J, Japanese version of the Impact of Event Scale-Revised; CATS, Child Abuse and Trauma Scale; NDI, neurite density index; ODI, orientation dispersion index; NVI, negative peak potential preceding the PVI wave of ABR; PVI, positive peak potential of the VI wave of the ABR; NVII, negative peak potential preceding the PVII wave of ABR; PVII, positive peak potential of the VII wave of the ABR; ΔVI, peak-to-peak potential of NVI and PVI; ΔVII, peak-to-peak potential of NVII and PVII.

### 3.5 Identification of neuroanatomical predictors of ABR amplitudes

**Table 3** summarizes the results of multiple regression analyses assessing the associations between ABR amplitudes and regional microstructural indices, with adjustments for age and sex. ODI in the temporal cortex was a significant predictor of NVII amplitude (P3: β = −6.608, *P* = 0.001; P4: β = −7.235, *P* = 0.004) and PVII amplitude (P3: β = −7.474, *P* = 0.012; P4: β = −7.783, *P* = 0.014). In addition, thalamic ODI significantly predicted NVI amplitude (P3: β = −3.759, *P* = 0.010; P4: β = −3.257, *P* = 0.010). These findings suggest that increased dendritic orientation dispersion in the temporal cortex and thalamus is associated with reduced ABR amplitudes, indicating that regional microstructural complexity may affect brainstem-level auditory processing in individuals with ASD. Given the use of stepwise variable selection and the small sample size (N = 27), the identified predictors should be interpreted with caution, and the results require confirmation in larger, independent samples.

**Table 3:** Multiple regression analyses predicting abr amplitudes from regional microstructural indices in individuals with ASD and TD controls.

Variables	R^2^		Adjusted R^2^		*P*		β		SE		95% confidence interval				*P* value	
											Boot LLCI		Boot ULCI			
	P3	P4	P3	P4	P3	P4	P3	P4	P3	P4	P3	P4	P3	P4	P3	P4
NVI	0.340	0.304	0.220	0.178	0.049	0.080										
ODI temporal							−4.151	−4.498	3.947	4.032	−7.466	−7.827	6.513	6.283	0.181	0.162
ODI thalamus							−2.372	−2.094	1.719	1.843	−6.085	−5.993	0.635	1.221	0.193	0.284
Age							0.012	−0.026	0.043	0.052	−0.079	−0.129	0.096	0.070	0.790	0.606
Male sex							−0.005	−0.004	0.003	0.003	−0.012	−0.011	0.001	0.002	0.191	0.249
PVI	0.355	0.342	0.237	0.222	0.040	0.048										
ODI temporal							−4.776	−5.340	3.376	3.445	−9.164	−9.559	3.856	3.743	0.062	0.052
ODI thalamus							−2.552	−2.138	1.867	2.001	−6.467	−6.509	1.024	1.478	0.174	0.297
Age							−0.036	−0.070	0.052	0.057	−0.148	−0.188	0.053	0.025	0.465	0.232
Male sex							−0.004	−0.003	0.004	0.004	−0.012	−0.011	0.003	0.004	0.287	0.337
NVII	0.639	0.617	0.573	0.548	<0.001	<0.001										
ODI temporal							−6.608	−7.235	2.320	2.315	−9.709	−10.483	−0.301	−1.128	0.001^**^	0.004^**^
ODI thalamus							−3.759	−3.257	1.288	1.210	−6.525	−5.891	−1.399	−1.085	0.010^*^	0.010^*^
Age							−0.093	−0.122	0.054	0.048	−0.190	−0.213	0.027	−0.021	0.100	0.015^*^
Male sex							0.004	0.004	0.002	0.002	−0.002	−0.001	0.008	0.008	0.111	0.080
PVII	0.517	0.478	0.429	0.383	0.002	0.005										
ODI temporal							−7.474	−7.783	3.473	3.672	−17.864	−17.535	−2.562	−1.960	0.012^*^	0.014^*^
ODI thalamus							−4.653	−4.391	2.330	2.388	−9.382	−9.113	−0.201	0.096	0.058	0.097
Age							−0.106	−0.082	0.065	0.073	−0.235	−0.222	0.023	0.062	0.117	0.287
Male sex							0.004	0.004	0.003	0.003	−0.002	−0.003	0.009	0.011	0.177	0.290

Standardized beta coefficients (β), standard errors (SE), 95% bias-corrected and accelerated bootstrap confidence intervals (Boot LLCI, Boot ULCI), and *p* values are reported. Independent variables included ODI values in the temporal cortex and thalamus; covariates were age and sex. Separate models were constructed for each ABR wave (NVI, PVI, NVII, PVII) at P3 and P4 electrode sites.

*P < 0.05, **P < 0.01.

ABR, auditory brainstem responses; ASD, autism spectrum disorder; TD, typically developing; ODI, orientation dispersion index; NVI, negative peak potential preceding the PVI wave of ABR; PVI, positive peak potential of the VI wave of the ABR; NVII, negative peak potential preceding the PVII wave of ABR; PVII, positive peak potential of the VII wave of the ABR.

### 3.6 Model 1: mediation of the association between temporal ODI and autism traits by ABR amplitude

**Figure 2:**
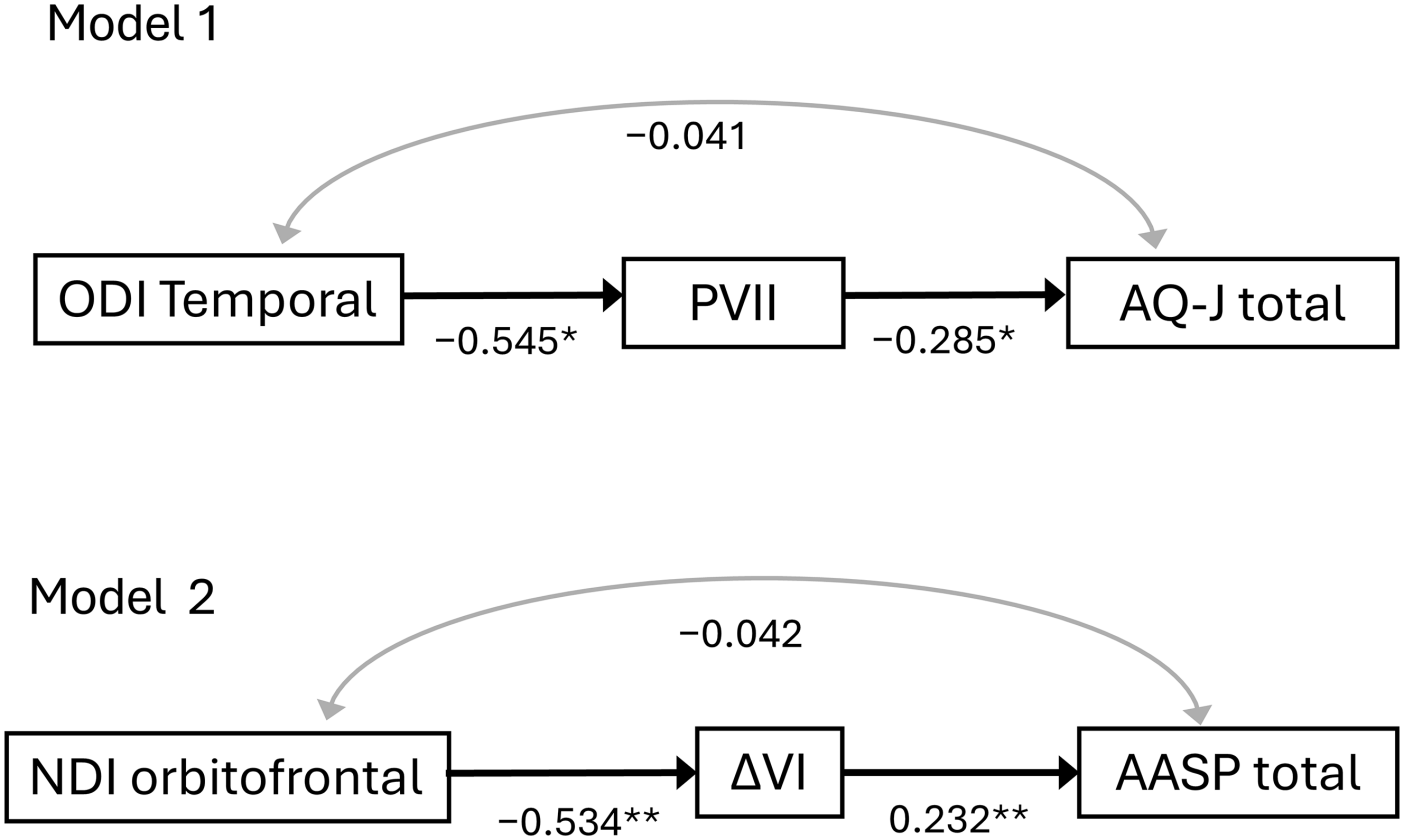
Path analysis model. Model 1, Mediation of the relationship between temporal cortical ODI and autism traits by PVII amplitude. The path analysis model illustrates the indirect statistical association between the orientation dispersion index (ODI) in the temporal cortex and autism symptom severity (AQ-J total score), with ABR wave PVII amplitude statistically accounting for this relationship. All pathways are adjusted for age and sex. Significant paths are shown in bold with standardized beta coefficients. *P < 0.05. AQ-J, Autism-Spectrum Quotient (Japanese version); PVII, positive peak of wave VII in the ABR. Model 2: Mediation of the relationship between orbitofrontal NDI and atypical sensory processing by ΔVI amplitude. The path analysis model illustrates the indirect statistical association between neurite density index (NDI) in the orbitofrontal cortex and atypical sensory processing difficulties (AASP total score), with ABR wave ΔVI amplitude statistically accounting for this relationship. All pathways are adjusted for age and sex. Significant paths are shown in bold with standardized beta coefficients. *P < 0.05, **P < 0.01. AASP, Adolescent/Adult Sensory Profile; ΔVI, peak-to-peak amplitude between NVI and PVI in the ABR.

A path analysis was conducted to examine whether PVII amplitude statistically accounted for the association between temporal cortex ODI and autism traits, given its significant associations with both temporal ODI and AQ-J scores. The model, adjusted for age and sex and estimated using maximum likelihood estimation (MLE), demonstrated excellent fit (χ²(6) = 1.21 × 10^-7^, *P* = 1.000; comparative fit index [CFI] = 1.252; root mean square error of approximation [RMSEA] = 0) (**Figure 2**; Model 1). As shown in **Supplementary Table 3**, temporal ODI was significantly associated with PVII amplitude (β = −0.545, *P* = 0.018), and PVII amplitude was significantly associated with AQ-J total scores (β = −0.285, P = 0.042). No direct association was found between temporal ODI and AQ-J scores, confirming a mediating effect of PVII amplitude. Further, **Table 4** shows that within the ASD group, PVII amplitude was significantly associated with AQ-J subscale scores for attention switching (P3: r = −0.782, *P* = 0.008; P4: r = −0.795, *P* = 0.006) and imagination (P3: r = −0.753, *P* = 0.015; P4: r = −0.686, *P* = 0.048). These findings suggest that auditory brainstem responses, particularly PVII, may represent a candidate association linking temporal cortical microstructure to core autism traits, including cognitive inflexibility and reduced imaginative capacity. Because this model was just-identified (saturated), conventional fit indices (CFI, RMSEA, χ²) are reported for descriptive purposes only and should not be interpreted as evidence of model validity; the apparently excellent fit reflects this saturation rather than empirical support for the specified causal structure. The path coefficients should therefore be regarded as descriptive estimates of association rather than as confirmation of the hypothesized mediation pathway.

**Table 4:** Partial correlations between AQ-J subscale scores and PVII amplitude in individuals with ASD, adjusted for age and sex.

AQ-J subscales	PVII	
	P3	P4
Social skill	−0.364	−0.354
Attention switching	−0.782^**^	−0.795^**^
Local details	−0.294	−0.222
Communication	−0.317	−0.327
Imagination	−0.753^*^	−0.686^*^

Analyses were conducted within the ASD group only. Spearman’s rank correlation coefficients (*r*) are reported for each AQ-J subdomain at P3 and P4 electrode sites.

*Adjusted *P* < 0.05, **Adjusted *P* < 0.01.

AQ-J, Japanese version of the Autism-Spectrum Quotient; ASD, autism spectrum disorder; PVII, positive peak potential of the VII wave of the ABR; ABR, auditory brainstem responses.

### 3.7 Model 2: mediation of the association between orbitofrontal NDI and atypical sensory processing by ABR ΔVI amplitude

A path analysis was conducted to examine whether ΔVI amplitude accounted for the association between OFC microstructure and atypical sensory processing, based on its significant negative correlation with NDI and moderate association with AASP total scores. The model, estimated using MLE and adjusted for age and sex, demonstrated adequate fit (χ²(6) = 0.408, *P* = 0.999; CFI = 1.121; RMSEA = 0) (**Figure 2**; Model 2). As shown in **Supplementary Table 4**, NDI in the OFC was significantly associated with ΔVI amplitude (β = −0.534, *P* = 0.003), which in turn was significantly associated with AASP total scores (β = 0.232, *P* = 0.002). No direct association was found between orbitofrontal NDI and AASP scores, consistent with, but not establishing, a statistical mediating role of ΔVI amplitude. **Table 5** presents partial correlations between ΔVI amplitude and AASP subscales in the ASD group. A significant positive correlation was observed with sensory avoiding behavior (AASP Sensation Avoiding: P3: r = 0.675, *P* = 0.045), particularly in the left hemisphere; the right hemisphere showed a similar but non-significant trend (P4: r = 0.520, *P* = 0.273). These findings suggest that reduced neurite density in the OFC may be associated with sensory avoidance behaviors in ASD, with ΔVI amplitude potentially accounting for part of this association. These results are descriptively consistent with a candidate brain–behavior pathway linking cortical microstructure to atypical sensory processing but do not establish such a pathway. As with Model 1, this model was just-identified (saturated); therefore, the reported fit indices (CFI, RMSEA, χ²) do not provide independent confirmation of the model and should not be interpreted as evidence of validation. The mediation findings should therefore be regarded as exploratory and hypothesis-generating.

**Table 5:** Partial correlations between AASP subscale scores and ΔVI amplitude in individuals with ASD, adjusted for age and sex.

AASP subscales	ΔVI	
	P3	P4
Low registration	0.395	0.376
Sensation seeking	0.269	0.347
Sensory sensitivity	0.499	0.343
Sensation avoiding	0.675^*^	0.520

Analyses were conducted within the ASD group only. Spearman’s rank correlation coefficients (*r*) are reported for each subscale at P3 and P4 electrode sites.

*Adjusted *P* < 0.05.

AASP, Adolescent/Adult Sensory Profile; ASD, autism spectrum disorder; ΔVI, peak-to-peak potential of NVI and PVI in the ABR; ABR, auditory brainstem responses.

### 3.8 Psychological predictors of OFC microstructure: a multiple regression analysis

To investigate whether psychological variables predict neurostructural properties in the OFC, multiple regression analyses were conducted with NDI and ODI as dependent variables, controlling for age and sex. As shown in **Table 6**, the model for NDI yielded a significant adjusted R² of 0.565 (*P* = 0.001), whereas the model for ODI showed no explanatory power (adjusted R² = −0.003, *P* = 0.467). Among the predictors, total scores for CATS (β = −4.501 × 10^-4^, *P* = 0.021) and IES-R (β = 5.372 × 10^-4^, *P* = 0.033) were significantly associated with orbitofrontal NDI. These results suggest that ACEs and trauma-related symptoms are significant psychological predictors of orbitofrontal microstructural alterations, as reflected by NDI, in individuals with ASD. As with the analyses in Section 3.5, this stepwise regression analysis should be regarded as exploratory, and the identified psychological predictors of orbitofrontal NDI should be validated in larger and demographically more balanced samples.

**Table 6:** Multiple regression analyses predicting NDI and ODI in the OFC in individuals with ASD and TD controls.

Variables	R^2^	Adjusted R^2^	*P* value	β	SE	95% confidence interval		*P* value
						Boot LLCI	Boot ULCI	
NDI orbitofrontal	0.682	0.565	0.001^*^					
AQ-J total				1.960×10^−4^	3.071 × 10^-4^	−4.351×10^−4^	7.603×10^−4^	0.512
Estimated IQ				−3.906×10^−5^	1.649 × 10^-4^	−3.548×10^−4^	3.002×10^−4^	0.793
AAPS total				−1.086×10^−4^	1.298 × 10^-4^	−3.711×10^−4^	1.501×10^−4^	0.398
CATS total				−4.501×10^−4^	1.936 × 10^-4^	−8.543×10^−4^	−6.115×10^−5^	0.021^*^
IES-R-J total				5.372×10^−4^	2.429 × 10^-4^	9.843×10^−5^	1.107×10^−3^	0.033^*^
Age				1.051×10^−3^	3.936 × 10^-4^	1.605×10^−4^	1.745×10^−3^	0.026^*^
Male sex				4.742×10^−3^	6.482 × 10^-^³	−7.792×10^−3^	1.829×10^−2^	0.390
ODI orbitofrontal	0.267	−0.003	0.467					

Standardized beta coefficients (β), standard errors (SE), *p* values, and 95% bootstrap confidence intervals (Boot LLCI, Boot ULCI) are reported.

Independent variables included psychological assessments (AQ-J, AASP, CATS, IES-R-J), estimated IQ, age, and sex.

*P < 0.05.

NDI, neurite density index; ODI, orientation dispersion index; AQ-J, Japanese version of the Autism-Spectrum Quotient; AASP, Adolescent/Adult Sensory Profile; IES-R-J, Japanese version of the Impact of Event Scale-Revised; CATS, Child Abuse and Trauma Scale; ASD, autism spectrum disorder; TD, typically developing; OFC, orbitofrontal cortex.

## 4 Discussion

The present study was conducted as a preliminary, exploratory investigation of whether individual differences in brain microstructure are associated with autism traits and atypical sensory processing and whether these associations may be mediated by ABR. Given the small and demographically imbalanced sample, the findings reported below are hypothesis-generating rather than confirmatory. Under forward masking conditions, we found that ODI in the temporal cortex was associated with PVII amplitude, which in turn was associated with autism traits as measured by the AQ-J. Similarly, reduced neurite density in the orbitofrontal cortex was associated with increased ΔVI amplitude, which in turn was associated with greater atypical sensory processing as indicated by AASP scores. Within the ASD group, lower PVII amplitude was correlated with greater difficulties in attention switching and imagination, whereas higher ΔVI amplitude was linked to sensory avoidance behaviors. Taken together, these preliminary findings tentatively suggest candidate auditory pathways through which regional brain microstructure may relate to clinical features of ASD. They should not be interpreted as evidence of causal mechanisms and require replication in larger, demographically matched, and pre-registered studies before any mechanistic conclusions can be drawn.

Later ABR waves, particularly waves VI and VII, may involve multiple central structures, including the thalamus and acoustic radiation. Previous studies have endeavored to ascertain the origins of this activity. For instance, Stockard and Rossiter report that wave VI originates from the medial geniculate body (MGB), whereas wave VII may originate from either the MGB or auditory radiation (59). Parkkonen, Fujiki & Mäkelä (60) have proposed that waves VI and VII may be indicative of MGB activity; however, this hypothesis has not yet been substantiated with conclusive evidence (61). Based on this background, we focused on whether individual differences in brain microstructure, specifically in the thalamus and temporal cortex, are associated with the amplitude of later ABR components, such as PVII. These regions are part of the auditory thalamocortical pathway and were selected as candidate areas that may be associated with brainstem-level auditory responses. It should be noted, however, that the anatomical sources of waves VI and VII remain incompletely understood and are inferred from theoretical models rather than from direct recordings of specific brain structures; the brain–ABR associations discussed below should therefore be interpreted with this caveat in mind.

Our analysis showed that ODI in both the thalamus and temporal cortex was significantly negatively correlated with the amplitude of PVII. When we tested both regions together, only temporal cortex ODI remained a significant predictor. This suggests that while both structures relate to PVII, the temporal cortex may have a more direct role. These findings may reflect modulation of later ABR waves by regional microstructural properties. The trend observed in this study aligns with the findings of Akiyoshi and Kaga, who noted that the waveform characteristics of waves VI and VII may vary owing to the MGBs being composed of multiple nuclei, each projecting to distinct cortical regions (62). In comparison with the findings of this study, it is plausible that the VI and VII waves may involve contributions from thalamocortical networks, as suggested by the spatial specificity of ODI correlations. Taken together, these findings suggest that structural variability in the temporal cortex may modulate later ABR waves, particularly PVII, through thalamocortical pathways.

This study demonstrated that variability in temporal cortical microstructure, particularly in dendritic orientation, was associated with reduced PVII amplitude in the ABR. This reduction in neural response statistically accounted for the association between temporal cortical microstructure and specific autism traits, particularly attention switching and imagination, as measured by the AQ-J. Within the ASD cohort, these two behavioral domains were also significantly correlated, suggesting shared cognitive underpinnings. These findings support the hypothesis that disorganized dendritic architecture in the temporal cortex disrupts auditory signal modulation via thalamic input, particularly in conditions requiring suppression of irrelevant stimuli, such as forward masking. Supporting this interpretation, our previous research demonstrated that individuals with ASD showed significantly higher ODI in the left occipital gyrus (OG), and that functional connectivity values between the left OG and the left middle temporal gyrus were significantly negatively associated with ADOS scores (63). Animal and cellular studies have shown increased complexity and disorganization of dendritic branching in BTBR mice, an established ASD model (64). Similar abnormalities, including excessive branching and misorientation, have also been reported in donor-derived iPS cells with *SHANK2* mutations and in neurons of *SHANK2* knockout rats (65, 66). These structural alterations may be associated with impaired filtering of sensory input. Furthermore, the neural underpinnings of auditory hypersensitivity and challenges in auditory filtering in ASD have been attributed to an increase in endogenous neural noise and disruption of the excitation–inhibition balance (67, 68). The observed reduction in PVII amplitude may thus reflect altered sensory processing dynamics resulting from such microstructural and functional changes.

In this study, lower NDI in the OFC was associated with a greater amplitude difference between NVI and PVI (ΔVI), an ABR component reflecting early sensory processing. This ΔVI amplitude statistically accounted for the relationship between OFC microstructure and overall atypical sensory processing, as measured by the AASP total score. Within the ASD group, ΔVI was particularly elevated in individuals with greater sensory avoidance, as reflected in the AASP Sensation Avoiding subscale. The OFC has been implicated in the selective suppression of sensory input through its reciprocal connections with the TRN, which modulates thalamocortical transmission. In a functional coupling analysis, Green et al. (7) reported that connectivity between the prefrontal and association cortices was enhanced in neurotypical children, whereas connectivity with sensory cortices was suppressed. In primates, the OFC receives substantial input from the dorsomedial thalamus and is interconnected with systems involved in emotion and reward, including the medial prefrontal cortex, cingulate gyrus, amygdala, hypothalamus, and nucleus accumbens. These anatomical and functional properties suggest that the OFC plays a key role in multisensory integration and behavioral regulation. Although our findings are indirect, they support a potential role of OFC microstructure in regulating sensory input in ASD.

MRI and comparative tissue studies in both humans and marmosets have demonstrated that NDI serves as an indicator associated with myelination and neurite density (69, 70). As previous research indicates that myelination in the prefrontal cortex is social experience-dependent, especially during the juvenile stage (71, 72), we investigated the associations between childhood experiences and NDI scores in the prefrontal cortex. We also examined these associations because our previous findings demonstrated correlations between childhood experiences and sensory behaviors in individuals with ASD and TD controls (73). Notable, the current study identified a significant association between CATS scores for ACEs and NDI values for the OFC. These findings are also largely consistent with previous human postmortem studies, which indicate that ACEs influence the number of oligodendrocytes in the ventromedial prefrontal cortex and the expression of myelin-related genes, suggesting that myelination in the prefrontal cortex depend on juvenile social- experience (74). From this perspective, reduced NDI in the OFC in the current study may indicate impaired myelination, potentially disturbing neural conduction and leading to atypical sensory processing. It should be emphasized, however, that NODDI-derived indices such as ODI and NDI indirectly reflect microstructural properties rather than serving as direct measures of neuronal connectivity or function. The present interpretations are therefore inferential and require confirmation using complementary techniques.

This study has several limitations. First, the sample size was relatively small, and the ASD and TD groups differed in age and sex distribution. These factors might have reduced statistical power to detect both within-group associations and subtle group differences in ABR amplitudes and NODDI-derived indices. In particular, the TD group consisted exclusively of male participants, whereas the ASD group included six female participants, resulting in partial confounding among group, sex, and age. Because the covariate distribution was not adequately sampled across these dimensions, statistical adjustment for age and sex was insufficient to fully resolve this imbalance. Accordingly, the mediation and path models reported here should be interpreted with particular caution, and the absence of significant group differences should not be interpreted as evidence of equivalence between groups. These sampling limitations should be considered when interpreting the robustness and external validity of the mediation and brain–behavior findings, which should be regarded as preliminary and hypothesis-generating. Future studies with larger, demographically matched samples are needed to confirm the generalizability of these findings. Second, the mediation/path models reported in this study (Models 1 and 2) were just-identified (saturated), with zero degrees of freedom. Under this specification, global fit indices (CFI, RMSEA, χ²) are mathematically constrained to indicate perfect fit and therefore do not constitute evidence of model validity; the reported fit statistics reflect saturation rather than empirical confirmation of the hypothesized causal structure. Combined with the small and demographically imbalanced sample, these limitations indicate that the mediation results should be interpreted as preliminary and exploratory and require confirmation in larger, over-identified models with independent samples. Third, the multiple regression analyses identifying neuroanatomical predictors of ABR amplitudes (Section 3.5) and psychological predictors of orbitofrontal microstructure (Section 3.8) employed a stepwise variable-selection procedure. In a sample of this size, stepwise regression is vulnerable to overfitting, unstable variable inclusion across resamples, and inflated nominal significance levels; the predictors identified through these procedures should therefore be regarded as exploratory and hypothesis-generating rather than confirmatory evidence of specific brain–behavior associations and should be re-examined in larger, independent samples using pre-specified models. Fourth, because of the cross-sectional design, causal inferences regarding the relationship between brain microstructure, auditory processing, and behavioral traits cannot be established. Fifth, the use of self-report questionnaires to assess clinical characteristics may introduce reporting bias or subjectivity. This concern is particularly relevant in individuals with ASD, in whom difficulties with introspection, alexithymia, and atypical interoceptive awareness may further compromise the validity of self-report measures. Future studies should therefore complement self-report instruments with informant-based ratings and objective behavioral or physiological measures.

## 5 Conclusion

In this preliminary, exploratory study, individual differences in brain microstructure, as measured by ODI in the thalamus and temporal cortex, were tentatively associated with ABR under forward-masking conditions in individuals with ASD. Notably, reduced ODI in these regions was associated with lower ABR amplitudes (peaks VI and VII), which in turn were associated with autism traits and atypical sensory processing; these associations are correlational and do not imply a directional or causal sequence. Furthermore, reduced neurite density in the OFC was associated with increased ABR ΔVI amplitude, which was statistically linked to greater sensory sensitivity. This microstructural variation was also associated with ACEs. Given the small and demographically imbalanced sample, these findings are best regarded as preliminary and hypothesis-generating and suggest candidate neural pathways through which brain structure may relate to auditory processing and behavioral features in ASD. Larger, demographically matched, and pre-registered confirmatory studies are warranted before any clinical or interventional implications are drawn.

## Data Availability

The raw data supporting the conclusions of this article will be made available by the authors, without undue reservation.
