# Supplementary material for: Linking auditory brain responses to cortical microstructure and sensory behaviors in autism spectrum disorder: a preliminary study
Source: Front Psychiatry. 2026 Jul 24;17:1688324. doi: 10.3389/fpsyt.2026.1688324 (PMC13447278; doi:10.3389/fpsyt.2026.1688324)
Supplement: Supplementary file 1 [file Table1.docx]

Supplementary Material

# 1 Supplementary Figures and Tables

**Supplementary Table S1. Group Comparisons of ABR Amplitudes between Individuals with ASD and Typically Developing Controls, Using Age and Sex as Covariates.**

|  |  | P3 | | | | | | | | |  | | P4 | | | | | | | | |
| --- | --- | --- | --- | --- | --- | --- | --- | --- | --- | --- | --- | --- | --- | --- | --- | --- | --- | --- | --- | --- | --- |
|  |  | TD | |  | ASD | |  | ANCOVA | | |  | TD | | |  | ASD | |  | ANCOVA | | |
|  |  | Average | SD |  | Average | SD |  | F |  | Adjusted *P* |  | Average | | SD |  | Average | SD |  | F |  | Adjusted *P* |
| NVI |  | -0.018 | 0.155 |  | 0.035 | 0.113 |  | 0.588 |  | 0.902 |  | −0.015 | | 0.139 |  | 0.023 | 0.116 |  | 0.006 |  | 1.000 |
| PVI |  | 0.008 | 0.147 |  | 0.096 | 0.129 |  | 0.680 |  | 0.836 |  | 0.014 | | 0.149 |  | 0.089 | 0.155 |  | 0.095 |  | 1.000 |
| NVII |  | −0.168 | 0.171 |  | −0.161 | 0.128 |  | 0.020 |  | 1.000 |  | −0.147 | | 0.174 |  | −0.153 | 0.144 |  | 0.088 |  | 1.000 |
| PVII |  | −0.036 | 0.220 |  | −0.028 | 0.164 |  | 0.001 |  | 1.000 |  | −0.001 | | 0.218 |  | −0.022 | 0.172 |  | 0.111 |  | 1.000 |
| ΔVI |  | 0.022 | 0.035 |  | 0.038 | 0.044 |  | 0.230 |  | 1.000 |  | 0.023 | | 0.043 |  | 0.044 | 0.043 |  | 0.757 |  | 0.787 |
| ΔVII |  | 0.132 | 0.100 |  | 0.133 | 0.117 |  | 0.053 |  | 1.000 |  | 0.146 | | 0.114 |  | 0.131 | 0.129 |  | 0.025 |  | 1.000 |

Note*:* Mean peak amplitudes (in µV) and standard deviations are shown for each waveform component recorded at P3 and P4 electrode sites. Statistical comparisons were performed using analysis of covariance (ANCOVA), controlling for age and sex. No significant group differences were observed after the Bonferroni correction.

TD, typically developing; SD, standard deviation; ASD, autism spectrum disorder; NVI, negative peak potential preceding the PVI wave of ABR; PVI, positive peak potential of the VI wave of the ABR; NVII, negative peak potential preceding the PVII wave of ABR; PVII, positive peak potential of the VII wave of the ABR; ΔVI, peak-to-peak potential of NVI and PVI; ΔVII, peak-to-peak potential of NVII and PVII; ABR, auditory brainstem responses.

**Supplementary Table S2. ROI-based Comparisons of NDI and ODI Values between Individuals with ASD and Typically Developing Controls, Controlling for Age and Sex.**

|  | TD | |  | ASD | |  | ANCOVA | |
| --- | --- | --- | --- | --- | --- | --- | --- | --- |
|  | Average | SD |  | Average | SD |  | F | Adjusted *P* |
| NDI temporal | 0.463 | 0.017 |  | 0.448 | 0.012 |  | 0.136 | 1.000 |
| NDI thalamus | 0.622 | 0.035 |  | 0.602 | 0.046 |  | 1.758 | 0.594 |
| NDI orbitofrontal | 0.485 | 0.014 |  | 0.473 | 0.015 |  | 0.338 | 1.000 |
| ODI temporal | 0.493 | 0.017 |  | 0.498 | 0.006 |  | 0.421 | 1.000 |
| ODI thalamus | 0.342 | 0.014 |  | 0.339 | 0.015 |  | 0.133 | 1.000 |
| ODI orbitofrontal | 0.458 | 0.016 |  | 0.461 | 0.011 |  | 1.156 | 0.880 |

Note*:* Mean NDI and ODI values (± SD) are reported for each region of interest (ROI): temporal cortex, thalamus, and orbitofrontal cortex. Statistical comparisons were conducted using analysis of covariance (ANCOVA) with age and sex as covariates. No significant group differences were observed after the Bonferroni correction.

TD, typically developing; SD, standard deviation; ASD, autism spectrum disorder; NDI, neurite density index; ODI, orientation dispersion index.

**Supplementary Table S3. Path Analysis Results for Model 1: Mediation of the Relationship between Temporal ODI and Autism Traits by PVII Amplitude.**

|  |  |  | Standardized β | 95% confidence interval | | *P* value |
| --- | --- | --- | --- | --- | --- | --- |
|  |  |  |  | Boot LLCI | Boot ULCI |  |
| AQ-J total | - | PVII P4 | −0.285 | −34.948 | −0.781 | 0.042^*^ |
| PVII P4 | - | ODI temporal | −0.545 | −20.016 | −3.306 | 0.018^*^ |
| ODI temporal | - | AQ-J total | −0.041 | −0.036 | 0.024 | 0.735 |

Note*:* Standardized beta coefficients (β), *p* values, and 95% bootstrap confidence intervals (Boot LLCI, Boot ULCI) are shown.

The model was adjusted for age and sex.

* *P* < 0.05.

AQ-J, Japanese version of the Autism-Spectrum Quotient; PVII, positive peak potential of the VII wave of the ABR; ODI, orientation dispersion index; ABR, auditory brainstem responses.

**Supplementary Table S4. Path Analysis Results for Model 2: Mediation of the Relationship between Orbitofrontal NDI and Sensory Processing Atypicalities by ΔVI Amplitude.**

|  |  |  | Standardized β | 95% confidence interval | | *P* value |
| --- | --- | --- | --- | --- | --- | --- |
|  |  |  |  | Boot LLCI | Boot ULCI |  |
| AASP total | - | ΔVI P3 | 0.232 | 0.063 | 550.511 | 0.002^**^ |
| ΔVI P3 | - | NDI orbitofrontal | −0.534 | −2.677 | −0.477 | 0.003^**^ |
| NDI orbitofrontal | - | AASP total | −0.042 | −0.144 | 0.073 | 0.630 |

Note*:* Standardized beta coefficients (β), *p* values, and 95% bootstrap confidence intervals (Boot LLCI, Boot ULCI) are reported.

The model was adjusted for age and sex.

* *P* < 0.05, ** *P* < 0.01.

AASP, Adolescent/Adult Sensory Profile, ΔVI, peak-to-peak potential of NVI and PVI in the ABR; NDI, neurite density index; ABR, auditory brainstem responses.
